# Association between prognostic nutritional index and 90-day readmission in patients with acute exacerbation of chronic obstructive pulmonary disease: a multicenter study

**DOI:** 10.3389/fnut.2026.1835781

**Published:** 2026-08-25

**Authors:** Yong Jiang, Hongmei Liu, Bicui Liu

**Affiliations:** 1 Department of Respiratory, Bishan Hospital of Chongqing Medical University, Chongqing, China; 2 Department of Respiratory and Critical Care Medicine, Bishan Hospital of Chongqing Medical University, Chongqing, China

**Keywords:** chronic obstructive pulmonary disease (COPD), prognostic factors, prognostic nutritional index (PNI), readmission, short-term readmission due to acute exacerbation

## Abstract

**Background:**

Chronic obstructive pulmonary disease (COPD) is marked by chronic airway inflammation and obstructive ventilation dysfunction. The prognostic nutritional index (PNI), an indicator for evaluating nutritional and immune status with confirmed prognostic value in most diseases, has an unclear association with short-term acute re-exacerbation of COPD. This study thus investigated the correlation between PNI levels and 90-day acute re-exacerbation in COPD patients.

**Methods:**

A cohort analysis was conducted on AECOPD patients, who were grouped by PNI tertiles. The primary outcome was 90-day readmission for acute re-exacerbation. Cox proportional hazards regression, Kaplan–Meier survival curves (Log-rank test), restricted cubic spline plots and subgroup analysis were applied for statistical assessment and conclusion verification.

**Results:**

A total of 112 patients had acute re-exacerbation. Fully adjusted multivariate Cox regression identified PNI as a protective factor (HR = 0.914, 95% CI: 0.877 ~ 0.952, *p* < 0.001). Restricted cubic spline analysis confirmed a linear dose–response between PNI and 90-day readmission risk (overall *p* = 0.0002, non-linear *p* = 0.737), with an inflection point at PNI = 43.05. The cumulative incidence of readmission showed a significant difference across PNI tertile groups (Log-rank *p* = 0.003). Subgroup analysis revealed consistent protective trends across all subgroups, with no significant interaction effects detected (all interaction *p* > 0.05).

**Conclusion:**

PNI is an independent protective factor for short-term readmission in AECOPD patients, with a significant linear dose–response relationship and a clinical threshold of PNI = 43.05.

## Introduction

Chronic obstructive pulmonary disease (C OPD) is a respiratory disease characterized by chronic airway inflammation and persistent airflow limitation (1). Acute exacerbation is a critical clinical event in the course of COPD, imposing a heavy medical burden on patients. It not only further impairs pulmonary function and reduces quality of life (2–4) but also may be life-threatening in severe cases (5). Therefore, early identification of patients at high risk of frequent acute exacerbations in the short term is of great clinical significance for improving prognosis.

COPD is highly heterogeneous. In addition to affecting the airways and lung parenchyma, it can impact multiple systems such as the heart and nutritional status through the inflammatory “spillover effect” (6), suggesting that clinical practice should focus not only on local pulmonary inflammation but also on the early intervention of extrapulmonary abnormalities.

Malnutrition is one of the common systemic complications of COPD. An incomplete statistical analysis showed that approximately 30.0% of COPD patients meet the diagnostic criteria for malnutrition, and half of them have nutritional risk (7), indicating that malnutrition is very prevalent in COPD. Moreover, malnutrition can exacerbate respiratory dysfunction. Previous studies have demonstrated that malnutrition leads to a decrease in diaphragmatic mass and thickness, as well as respiratory muscle strength and endurance; diaphragmatic weakness may increase the risk of progression to respiratory failure in COPD (8–10) and is closely associated with an elevated risk of death in patients. Lara Hersberger et al. conducted an observational study on 2028 patients and found a close relationship between malnutrition and the risk of death (11). Nicolaas E Deutz et al. performed a randomized controlled trial involving 652 patients and concluded that nutritional support can significantly reduce the risk of death in patients (12).

Existing studies have confirmed that malnutrition increases the risk of readmission in COPD patients. Body mass index (BMI), a commonly used nutritional assessment indicator, is also considered a risk factor for long-term readmission (13), and a multicenter study also found that BMI is an important factor for readmission due to acute exacerbation (14). However, the predictive efficacy of a single indicator for prognostic risk is limited, so more comprehensive and simple composite indicators are still needed to fully evaluate the readmission risk of COPD patients.

The prognostic nutritional index (PNI), calculated from serum albumin and lymphocyte count, can comprehensively assess the nutritional and immune status of patients and has been proven to be closely associated with adverse prognosis in multiple chronic diseases (15–17, 32). Currently, no study has specifically examined the association between PNI and 90-day hospital-verified re-hospitalization in AECOPD patients.

Therefore, this study aims to investigate the relationship between PNI and short-term readmission due to acute re-exacerbation in AECOPD patients after discharge, and to clarify its clinical predictive value.

## Data sources and methods

### Data sources

**Figure 1:**
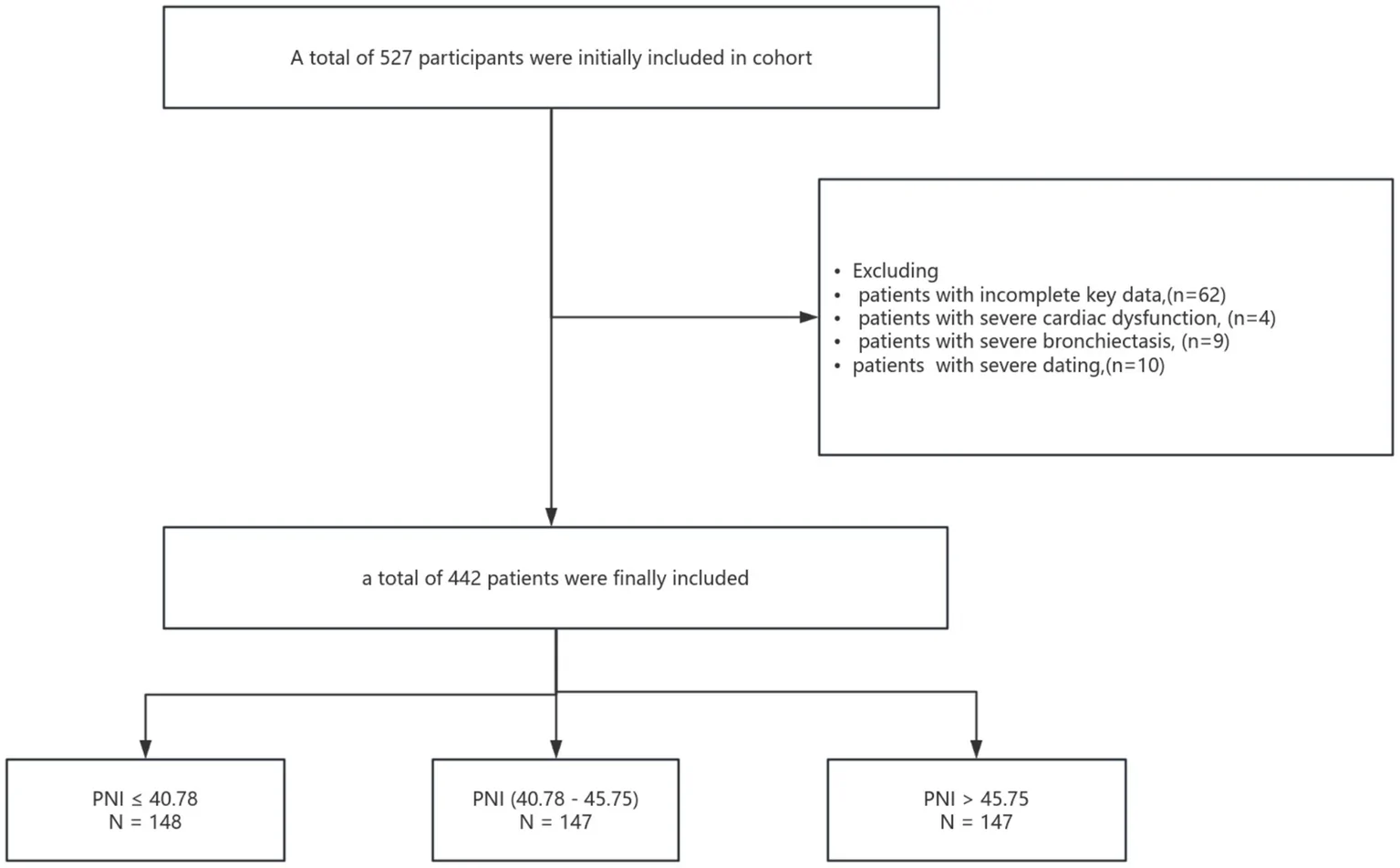
Described the inclusion and exclusion process.

Data for this study were obtained from the retrospective collection of medical records from the Affiliated Bishan Hospital of Chongqing Medical University and Bishan District Hospital of Traditional Chinese Medicine from January 2019 to June 2024. A total of 527 patients who regularly followed up at the hospital outpatient clinic with inhalant preparations were initially included. After exclusion of 72 patients with incomplete key data, 4 patients with severe cardiac insufficiency, 9 patients with severe bronchiectasis, and 10 patients with severe missing data, 442 patients were finally enrolled (Figure 1).

Inclusion criteria for COPD patients: (1) Only patients who were hospitalized for the first time due to AECOPD in the Department of Respiratory and Critical Care Medicine during follow-up; (2) Aged 40–80 years.

Exclusion criteria: Complicated with asthma, malignancy, interstitial lung disease, severe bronchiectasis, missing serum albumin or lymphocyte data, less than two regular outpatient follow-ups within 90 days in medical records, and no history of inhalant use.

### Data extraction and PNI calculation

Data were collected from the Affiliated Bishan Hospital of Chongqing Medical University and Bishan District Hospital of Traditional Chinese Medicine, including demographics, laboratory parameters, and comorbidities. All data were collected within 24 h of admission to ensure consistency. Demographic variables included age, gender, height, weight, and smoking status. Comorbidities included hypertension, coronary heart disease, diabetes mellitus, and cerebral infarction. Laboratory parameters included oxygenation status, pH, neutrophils, lymphocytes, eosinophil percentage, hemoglobin, platelets, red blood cell distribution width, blood urea nitrogen (BUN), creatinine (Cr), uric acid, albumin, high-density lipoprotein (HDL), low-density lipoprotein (LDL), cholesterol, triglyceride, and the time of subsequent readmission.

The formula for calculating the prognostic nutritional index (PNI) is: PNI = (10 × serum albumin [g/dL]) + (0.005 × lymphocyte count [K/μL]).

Patients were equally divided into three groups (low, medium, high) according to the tertiles of PNI levels.

### Outcome definition

The primary outcome was readmission due to acute exacerbation within 90 days after discharge.

### Statistical analysis

For normally distributed data, continuous variables were expressed as mean ± standard deviation (SD); for non-normally distributed data (skewness ≥ 1 or ≤ − 1), they were expressed as median with interquartile range [IQR]. Parametric tests were used: ANOVA for normally distributed variables, and the Kruskal-Wallis H test for skewed distributions. Categorical variables were analyzed using the chi-square test. For missing data, multiple imputation using predictive mean matching (PMM) was performed with 5 imputed datasets and 10 iterations. This study adhered to the STROBE reporting guidelines.

Cox proportional hazards regression models were used for risk assessment, restricted cubic spline plots for dose–response evaluation, Kaplan–Meier curves for survival analysis, and the Log-rank test for assessing the incidence of 90-day acute exacerbation events across PNI groups. Three Cox models with increasing adjustment were constructed: Model 1 (age, sex); Model 2 (age, sex, BMI); Model 3 (age, sex, BMI, hypertension, coronary heart disease, diabetes, stroke, oxygenation index, C-reactive protein, creatinine). Bootstrap validation with 1,000 resamples was performed. Subgroup interaction tests served as the primary basis for interpretation.

All data analyses were performed using Rstudio version 4.5.2, with statistical significance defined as a two-sided *p* value <0.05.

## Results

A total of 442 subjects were enrolled and divided into three groups according to PNI levels: PNI ≤ 40.78 (*n* = 148), 40.78 < PNI ≤ 45.75 (*n* = 147), and PNI > 45.75 (*n* = 147). The results showed (Table 1) no statistically significant differences in sex, smoking, diabetes, cerebral infarction, prior one-year readmission, number of exacerbations, pH, monocytes, RDW, BUN, Cr, uric acid, or platelet count among the three groups (*p* > 0.05).

**Table 1:** Baseline comparison based on PNI tertiles.

Characteristic	PNI ≤40.78 (*n* = 148)	PNI 40.78–45.75 (*n* = 147)	PNI >45.75 (*n* **=** 147)	*P* value
Age	69.9 ± 6.6	70.9 ± 6.0	67.9 ± 6.7	<0.001
Gender				0.753
Female	28 (18.9%)	30 (20.4%)	33 (22.4%)	
Male	120 (81.1%)	117 (79.6%)	114 (77.6%)	
Smoking status	105 (71%)	100 (68%)	96 (65%)	0.583
BMI	19.8 [17.8, 22.7]	20.7 [18.4, 23.4]	21.5 [19.5, 24.2]	<0.001
Diabetes mellitus	16 (11%)	18 (12%)	16 (11%)	0.909
Stroke	6 (4.1%)	8 (5.4%)	5 (3.4%)	0.835
Hypertension	12 (8.1%)	12 (8.2%)	15 (10.2%)	0.770
Heart failure	30 (20.3%)	35 (23.8%)	14 (9.5%)	0.004
Coronary heart disease	34 (23.0%)	30 (20.4%)	13 (8.8%)	0.003
Number of exacerbations within 1 year	1.0 [0.0, 2.0]	1.0 [0.0, 2.0]	0.0 [0.0, 2.0]	0.285
PH	7.4 ± 0.1	7.4 ± 0.1	7.4 ± 0.0	0.897
Oxygenation index	293.3 ± 94.7	316.6 ± 99.8	318.2 ± 98.3	0.051
Neutrophil	7.3 [5.2, 10.2]	6.2 [4.2, 8.9]	5.4 [4.0, 7.6]	<0.001
Lymphocyte	0.7 [0.5, 1.0]	0.9 [0.7, 1.2]	1.4 [1.1, 1.8]	<0.001
Monocyte	0.6 [0.4, 0.8]	0.5 [0.4, 0.8]	0.5 [0.4, 0.7]	0.496
Eosinophil percentage	0.4 [0.1, 1.3]	0.8 [0.2, 2.3]	1.3 [0.2, 2.8]	<0.001
RDW	13.7 [13.1, 14.9]	13.5 [12.9, 14.4]	13.4 [12.8, 14.1]	0.054
Hemoglobin	133.3 ± 18.9	132.3 ± 22.9	140.1 ± 26.7	0.008
Platelet	201.7 ± 73.8	201.6 ± 76.6	212.0 ± 73.1	0.392
CRP	33.2 [9.6, 91.7]	12.1 [2.0, 45.1]	3.3 [0.5, 14.2]	<0.001
Albumin	33.7 ± 2.7	38.6 ± 2.2	41.5 ± 2.7	<0.001
BUN	5.6 [4.3, 8.3]	5.8 [4.5, 7.5]	5.5 [4.6, 6.8]	0.364
Cr	67.0 [54.0, 83.2]	64.0 [56.0, 77.5]	63.0 [53.0, 75.5]	0.402
Uric acid	299.8 ± 135.4	281.7 ± 120.6	273.7 ± 132.1	0.210
BNP	400.9 [145.9, 1594.2]	259.7 [114.6, 920.5]	116.5 [58.3, 248.8]	<0.001
HDL	1.2 [1.0, 1.4]	1.3 [1.1, 1.6]	1.4 [1.1, 1.7]	<0.001
LDL	2.1 ± 0.7	2.3 ± 0.8	2.6 ± 0.7	<0.001
Cholesterol	3.7 ± 0.9	4.2 ± 1.1	4.6 ± 1.0	<0.001
TG	0.8 [0.6, 1.1]	0.9 [0.7, 1.2]	1.1 [0.8, 1.4]	<0.001
90-day readmission	51 (34.5%)	35 (23.8%)	26 (17.7%)	0.004

Mean ± SD (normal); Median [IQR] (skewed); *n* (%). Kruskal-Wallis rank sum test; Pearson chi-square test.

The mean age of the PNI ≤ 40.78 group was higher (69.9 ± 6.6 years) than the PNI > 45.75 group (67.9 ± 6.7 years) (*p* < 0.001). BMI changed synchronously with PNI grouping (*p* < 0.001). The prevalence of heart failure (*p* = 0.004) and coronary heart disease (*p* = 0.003) was significantly higher in the low PNI group.

In terms of laboratory parameters, oxygenation index, lymphocyte count, eosinophil percentage, hemoglobin, albumin, HDL, LDL, cholesterol, and triglyceride all showed an increasing trend with the elevation of PNI, while neutrophils, C-reactive protein (CRP), and brain natriuretic peptide (BNP)showed a gradual decreasing trend with the elevation of PNI; all the above indicators had statistically significant intergroup differences (*p* < 0.001). Red blood cell distribution width showed no significant difference among groups (*p* = 0.062). Platelet count showed an increasing trend with the elevation of PNI, but the intergroup difference was not statistically significant (*p* = 0.392).

The readmission rate showed a clear decreasing trend with increasing PNI tertile: T1 34.5%, T2 23.8%, T3 17.7%, with a statistically significant difference (*p* = 0.004).

## Clinical outcomes

### Cox proportional hazards analysis

Cox regression showed (Table 2): Model 1 HR = 0.910 (95%CI: 0.876–0.945); Model 2 HR = 0.913 (95%CI: 0.878–0.949); Model 3 HR = 0.914 (95%CI: 0.877–0.952), all *p* < 0.001. Bootstrap (1,000 reps) stability analysis: median HR = 0.912, 95%CI [0.872, 0.954].

**Table 2:** Results of proportional hazards regression analysis on the association between PNI and readmission.

Model	HR	95%CI	*P* value
Model 1	0.910	0.876 ~ 0.945	<0.001
Model 2	0.913	0.878 ~ 0.949	<0.001
Model 3	0.914	0.877 ~ 0.952	<0.001

Model 1: Adjusted for age and gender.

Model 2: Adjusted for age, gender and BMI.

Model3: Adjusted for age, gender, BMI, hypertension, CHD, diabetes, stroke, oxygenation, CRP and creatinine.

### Dose–response relationship analysis based on restricted cubic spline

**Figure 2:**
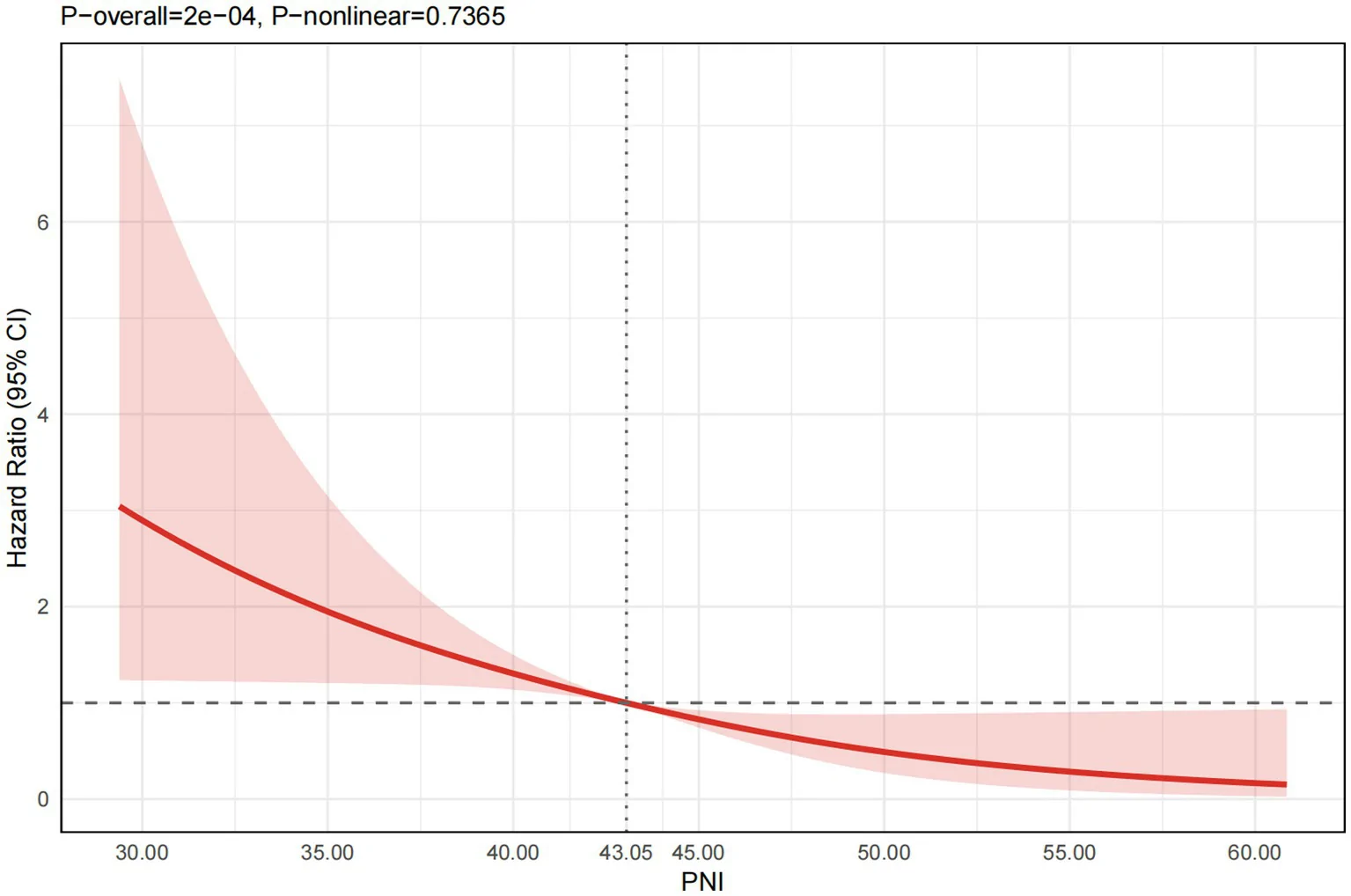
Restricted cubic spline curve show the association between PNI and readmission, adjusted for age, gender, BMI, hypertension, CHD, diabetes, stroke, oxygenation, CRP, and creatinine.

Under the full adjustment of Model 3 (Figure 2), the RCS plot showed a significant overall association (overall *p* = 0.0002) with no statistically significant non-linear relationship (non-linear *p* = 0.7365), indicating a linear dose–response relationship. The risk was lower when PNI was above 43.05 and increased when PNI was below 43.05.

### Survival analysis

**Figure 3:**
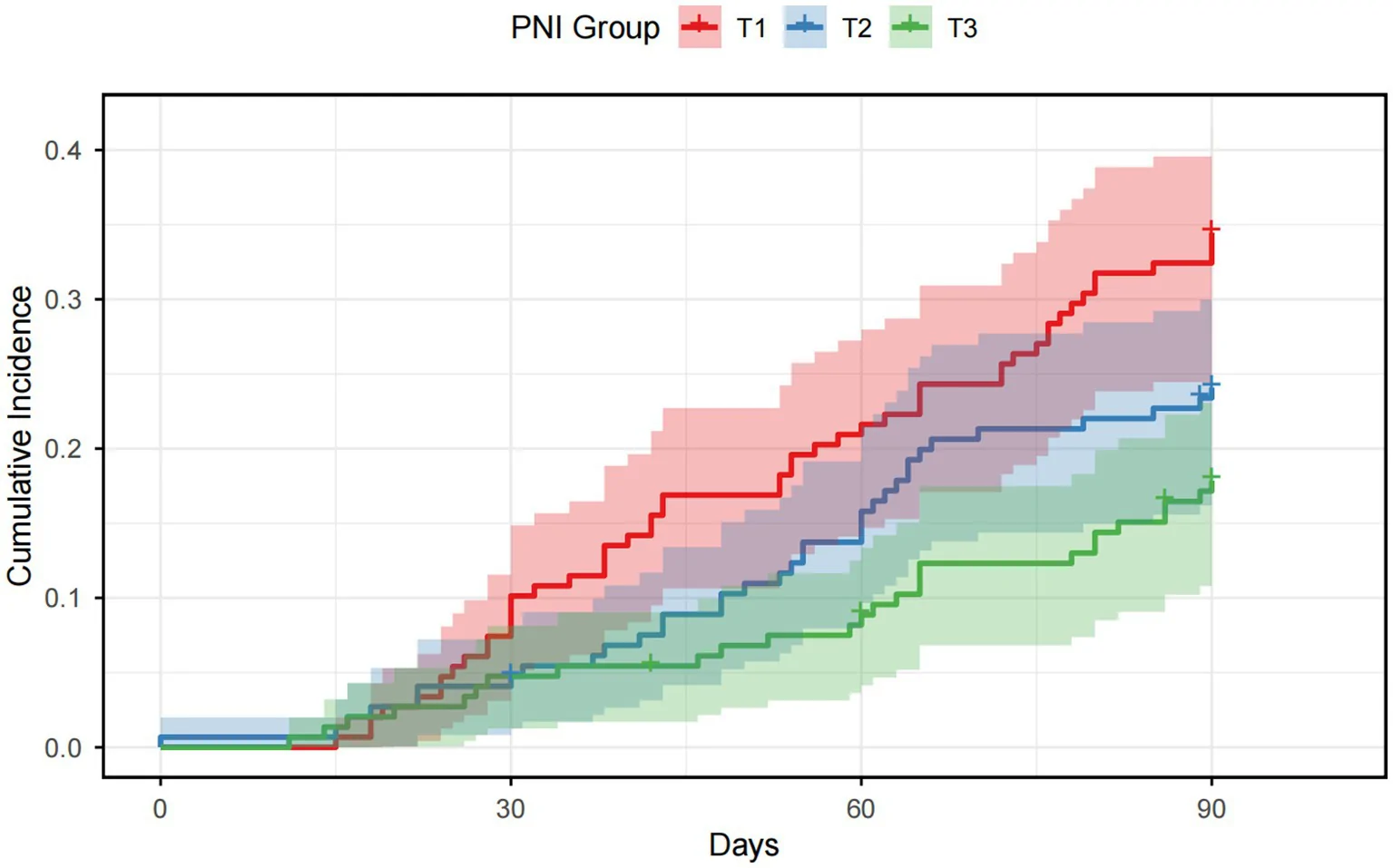
KM survival analysis plot showing the relationship between PNI and readmission.

The readmission rate decreased with increasing PNI tertile (Table 3): T1 34.5%, T2 23.8%, T3 17.7%. The Log-rank test indicated a significant difference in cumulative incidence among the groups (*p* = 0.003), as reflected in the Kaplan–Meier cumulative incidence plot (Figure 3).

**Table 3:** Number of readmissions at different PNI levels.

Group	Sample size	Readmissions	Readmission rate
T1	148	51	34.5%
T2	147	35	23.8%
T3	147	26	17.7%

T1 represents PNI ≤40.78. T2 represents PNI40.78–45.75. T3 represents PNI >45.7.

### Subgroup analysis

**Figure 4:**
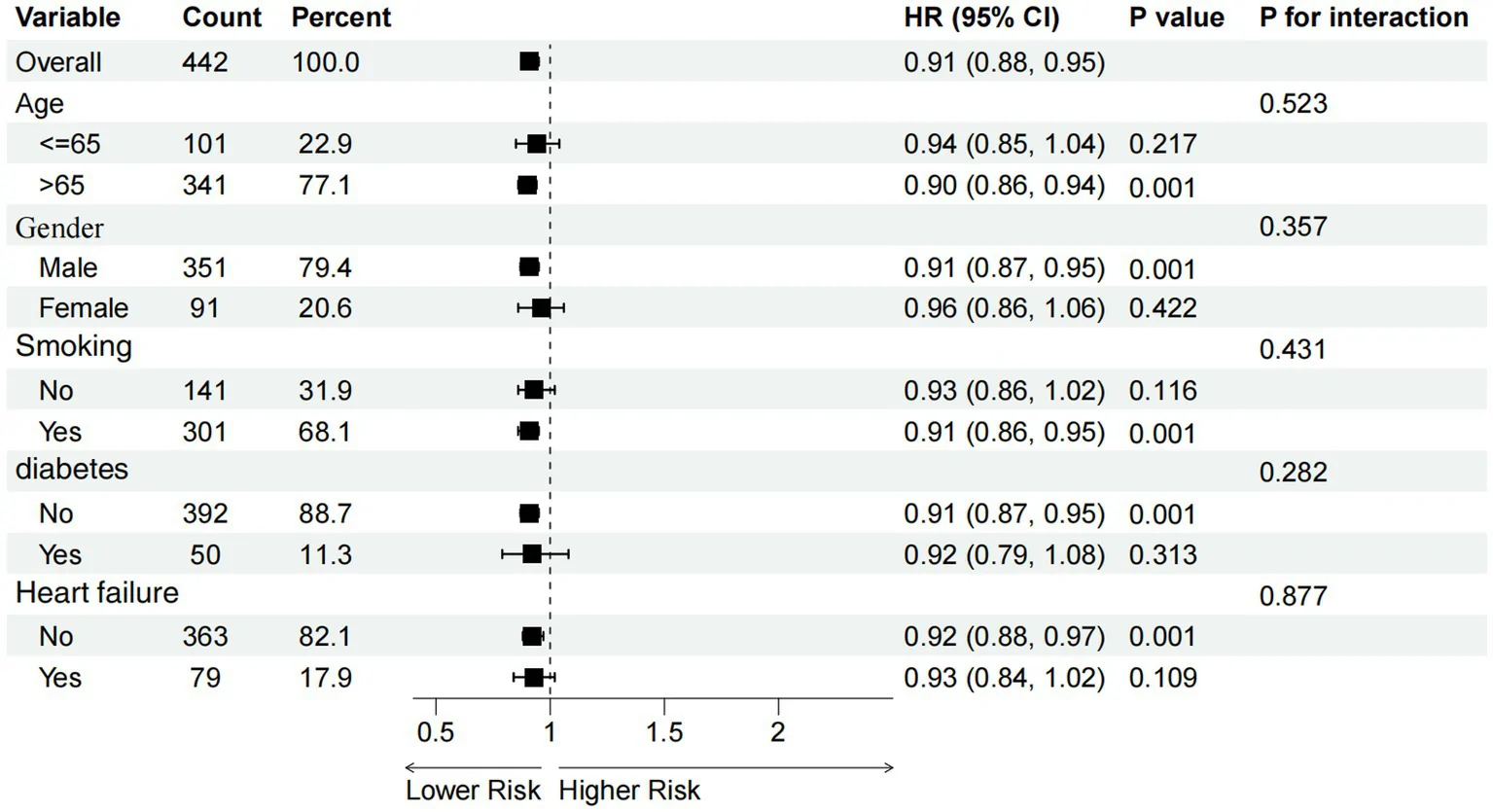
Subgroup analysis and random forest plot showing PNI and readmission.

Subgroup analysis (Figure 4) was performed by age (>65 years), sex, smoking, diabetes, and heart failure. The protective effect of PNI was consistent across all subgroups, with no significant interaction detected: age (*p* = 0.523), sex (*p* = 0.357), smoking (*p* = 0.431), diabetes (*p* = 0.282), heart failure (*p* = 0.877) (Table 4).

**Table 4:** Subgroup analysis.

Subgroup	HR (95% CI)	*P* value	Interaction *P* value
Age			0.523
≤65 years	0.94 (0.85–1.04)	0.217	–
>65 years	0.90 (0.86–0.94)	<0.001	–
Gender			0.357
Male	0.91 (0.87–0.95)	<0.001	–
Female	0.96 (0.86–1.06)	0.422	–
Smoking status			
No	0.93 (0.86–1.02)	0.116	–
Yes	0.91 (0.86–0.95)	<0.001	–
Diabetes			0.282
No	0.91 (0.87–0.95)	<0.001	–
Yes	0.92 (0.79–1.08)	0.313	–
Heart failure			0.877
No	0.92 (0.88–0.97)	0.001	–
Yes	0.93 (0.84–1.02)	0.109	–

## Discussion

This study investigated the relationship between PNI and 90-day re-hospitalization in AECOPD patients. Low PNI was associated with an increased risk of short-term re-hospitalization (HR = 0.914, 95%CI: 0.877 ~ 0.952, *p* < 0.001). RCS confirmed a linear dose–response relationship with a clinical reference value of PNI = 43.05.

As a nutritional assessment indicator, albumin also exerts an antioxidant effect (18), which can alleviate inflammatory damage to the body to a certain extent. The core pathological feature of COPD is chronic persistent inflammation of the airways and lung tissue, and chronic airway inflammation can lead to systemic chronic inflammation (19). The inflammatory state continuously consumes the body’s nutritional reserves and impairs anti-inflammatory capacity (20). Therefore, low albumin often indicates a poorer nutritional status and anti-inflammatory capacity, and poor nutritional status has a significant adverse impact on the prognosis of COPD patients, including reduced quality of life, deterioration of pulmonary function, disease exacerbation, and increased hospitalization frequency (7, 21, 22). Previous studies have found that COPD patients with hypoalbuminemia have an increased risk of death (23).

Lymphocyte count is an important indicator for evaluating the body’s immune function. Its level not only reflects immune defense capacity but also indirectly indicates nutritional status (24). Lower lymphocyte count may lead to a higher risk of infection (25), and infection is the primary inducement of acute exacerbation of COPD. Therefore, low lymphocyte count indirectly promotes short-term readmission of patients by increasing the risk of infection.

Thus, the interpretability of a single indicator is limited. The prognostic nutritional index, composed mainly of albumin and lymphocytes, comprehensively considers nutritional and immune status, enabling a more comprehensive and objective assessment of the prognostic risk of patients, which is the core advantage of PNI over a single nutritional or immune indicator in terms of clinical interpretability.

The prognostic nutritional index was first proposed by Buzby in 1980 and later modified by H. Onodera to assess the risks associated with postoperative mortality and complication incidence in patients with gastrointestinal tumors (26). Since then, its application scope has gradually expanded to prognostic assessment of various diseases. Existing evidence has shown that PNI is independently associated with adverse prognosis in tumors, autoimmune diseases, and heart diseases (15–17). In the respiratory field, PNI can be used as a prognostic management indicator in community-acquired pneumonia and COVID-19 (27, 28, 33), suggesting that PNI has broad applicability in the prognostic assessment of respiratory diseases.

Previous studies have explored the prognostic value of PNI in COPD. Fu-Zhen Yuan et al. reviewed data from 385 patients and found an association between PNI and adverse outcomes in COPD patients (29), the main results of that study focused on the risks of endotracheal intubation and transfer to the intensive care unit (ICU), without investigating short-term readmission in patient prognosis. In addition, PNI is also closely associated with short-term mortality in COPD (29–31).

This study has several strengths, as well as certain limitations. In terms of strengths, this study explored the linear association between PNI and short-term acute exacerbation in COPD patients, and further verified a clinically applicable risk cut-off value, with results having both statistical significance and clinical application value. In addition, the study strictly defined the study population through follow-up medication pickup records, effectively controlling the confounding bias caused by differences in medication adherence and improving the reliability of the results.

This study has several limitations. First, as a retrospective study, outcome events may be subject to passive capture by the medical records system. We enrolled only first-ever AECOPD admissions; for patients with frequent exacerbations, future multicenter prospective studies are needed. Second, post-discharge management factors such as pulmonary rehabilitation, discharge care intensity, and social support were not included in the analysis, which may introduce residual confounding.

In summary, this study confirmed that low PNI is an independent risk factor for short-term acute exacerbation and readmission in COPD patients after discharge, with a significant linear dose–response relationship and a clinical threshold of PNI = 43.05. As a simple, easily obtainable, and quantifiable composite indicator, PNI can effectively evaluate the nutritional and immune status of COPD patients, providing an important reference for clinical prediction of short-term prognosis, screening of high-risk patients, and formulation of individualized nutritional support and immunomodulatory intervention strategies.

## Conclusion

This study confirmed that low PNI is an independent risk factor for short-term acute exacerbation and readmission in COPD patients after discharge, with a significant linear dose–response relationship and a clinical threshold of PNI = 43.05. Further prospective, multicenter studies are still needed to verify these findings and explore whether early nutritional intervention in high-risk patients with low PNI can effectively reduce the risk of short-term readmission.

## Data Availability

The raw data supporting the conclusions of this article will be made available by the authors, without undue reservation.
